# *Lactobacillus* Decelerates Cervical Epithelial Cell Cycle Progression

**DOI:** 10.1371/journal.pone.0063592

**Published:** 2013-05-10

**Authors:** Katarina Vielfort, Linda Weyler, Niklas Söderholm, Mattias Engelbrecht, Sonja Löfmark, Helena Aro

**Affiliations:** 1 Department of Molecular Biosciences, The Wenner-Grens Institute, Stockholm University, Stockholm, Sweden; 2 Swedish Institute for Communicable Disease Control, Solna, Sweden; Mayo Clinic, United States of America

## Abstract

We investigated cell cycle progression in epithelial cervical ME-180 cells during colonization of three different *Lactobacillus* species utilizing live cell microscopy, bromodeoxyuridine incorporation assays, and flow cytometry. The colonization of these ME-180 cells by *L. rhamnosus* and *L. reuteri*, originating from human gastric epithelia and saliva, respectively, was shown to reduce cell cycle progression and to cause host cells to accumulate in the G1 phase of the cell cycle. The G1 phase accumulation in *L. rhamnosus*-colonized cells was accompanied by the up-regulation and nuclear accumulation of p21. By contrast, the vaginal isolate *L. crispatus* did not affect cell cycle progression. Furthermore, both the supernatants from the lactic acid-producing *L. rhamnosus* colonies and lactic acid added to cell culture media were able to reduce the proliferation of ME-180 cells. In this study, we reveal the diversity of the *Lactobacillus* species to affect host cell cycle progression and demonstrate that *L. rhamnosus* and *L. reuteri* exert anti-proliferative effects on human cervical carcinoma cells.

## Introduction

Non-keratinizing cervical epithelial cells of the human body are a well-functioning barricade against the outer surroundings, protecting the human body against harmful external agents and damage. To maintain the fidelity of this barrier, epithelial cells are renewed by cell division. The cell cycle is divided into four phases, gap 1 (G1), synthesis (S), gap 2 (G2) and mitosis (M), and the epithelium consists of cells continuously progressing through the four different cell cycle phases [1]. Cell cycle progression is driven by cyclin-dependent kinases (CDKs) and cyclins. The regulation of CDK-cyclin complex activity occurs through cyclin-dependent kinase inhibitors (CKIs), such as p21, at checkpoints that can halt cell cycle progression [2]. Cells may also cease active growth permanently or temporarily due to various influences, including contact inhibition and high cellular confluence; under these conditions, non-transformed cells enter a state of quiescence known as G0.

*Lactobacillus* bacteria, which encompass over 100 described species, are harmless extracellular lactic acid-producing inhabitants of the human body. The lower genital tract in healthy female individuals is dominated by *Lactobacillus* species at a level of 10^7^–10^8^ colony-forming units per gram of fluid [3]. The long chains of aggregated lactobacilli cover the epithelial cell layer as a protective coat, thereby contributing to the epithelial barrier. It is generally accepted that lactobacilli play a major role in maintaining urogenital health, given that the disruption of the population balance of these bacteria, or the depletion of vaginal lactobacilli, increases the incidence of bacterial vaginosis [4], [5].

It has also been demonstrated that certain *Lactobacillus* strains exert anti-proliferative effects on cancer cells [6], [7], [8]. The oral intake of *L. plantarum* reduced colon tumors in rats [9], and *L. rhamnosus* implantations in mice induced bladder tumor regression [10]. However, the molecular mechanism underlying these effects remains relatively unexplored. In this study, we have investigated the host cell cycle progression in cervical epithelial ME-180 cells upon colonization by three different *Lactobacillus* strains. We show that two out of the three *Lactobacillus* strains that were tested decelerate host cell proliferation and delay the host cells in the G1 phase of the cell cycle; moreover, we show that lactic acid production is a contributing factor to the observed cell cycle deceleration.

## Materials and Methods

### Cell Lines and Growth Conditions

The ME-180 (ATCC HBT-33) epithelial-like adenocarcinoma cell line from the human cervix was cultured in Dulbecco’s modified Eagle’s medium (DMEM) containing GlutaMAX (Invitrogen, Carlsbad, CA, USA) and supplemented with 10% fetal bovine serum (FBS) (Invitrogen). The cells were maintained at 37°C in 5% CO_2_. In all of the assays, a monolayer of cells that was 40–60% confluent was used.

### Bacterial Strains

The *Lactobacillus* strains used were originally isolated from healthy human individuals, and kindly provided by Stefan Roos and Hans Johansson of the Swedish Agricultural University of Science. *Lactobacillus rhamnosus* (Kx 151 A1) originated from a human gastric biopsy. *Lactobacillus reuteri* (FJ1) originated from human saliva. *Lactobacillus crispatus* (MV24-1a) originated from a human vagina. As described in a previously published study, these strains adhere to ME-180 cells [11]. The lactobacilli were cultured with no agitation in liquid MRS broth (Oxoid, Cambridge, UK) or on Rogosa agar plates (Oxoid, Cambridge, UK) at 37°C. None of the lactobacillus strains produced detectable levels of hydrogen peroxide. Overnight cultures of lactobacilli in liquid MRS broth were collected by centrifugation and washed once in DMEM prior to the assays that were performed in this study. The optical density at 600 nm of each bacterial sample was measured to calculate the number of bacteria that were present per milliliter. For the assays, the lactobacilli were added to cells in DMEM/10% FBS and incubated at 37°C in 5% CO_2_.

### Live-cell Time-lapse Imaging

ME-180 cells were grown overnight in 35 mm poly-D-lysine-coated glass-bottom dishes (MatTek Corp., Ashland, MA, USA) to a non-confluent monolayer. At the start of the assay, cells were washed once and then transferred to a live-cell incubator that was connected to an inverted microscope (Cell Observer, Carl Zeiss). The cells were maintained at 37°C in 5% CO_2_ throughout the microscopy process. Ten randomly selected positions (each containing 20–50 cells in the field of vision) were observed with a 20× objective for 16 hours. Differential interference contrast (DIC) images were taken every 10 minutes for each of the positions that were randomly chosen. Data were collected from three independent experiments, and a total of 3×300 cells were counted for each of the four types of conditions that were examined, namely, ME-180 cells alone and ME-180 cells that had been colonized with each of the three different *Lactobacillus* strains that were assessed in this study.

### The BrdU Proliferation Assay

Lactobacilli were added (multiplicity of infection 50) to non-confluent ME-180 cells. ME-180 cells with no bacteria added were used as a control and were normalized to 100% bromodeoxyuridine (BrdU) uptake. To determine if pH changes could influence cell proliferation, DMEM in which the pH had been reduced with lactic acid (Sigma-Aldrich) was added to the cells. In addition, the supernatants of lactobacilli after 24 hours of incubation with ME-180 cells was sterile filtered, measured for their pH changes, and added to new cells. The supernatant from cells that were incubated without the addition of bacteria was used as a control for this assay. The cells that had experienced the addition of bacteria, supernatant, or pH-reduced DMEM were all incubated for 24 hours. Two hours prior to the termination of the assay, BrdU was added, allowing the dye to be incorporated into the replicating DNA of the cells that were in S phase. To quantify the incorporation of BrdU into S phase cells, a BrdU ELISA kit (QIA58, Calbiochem, San Diego, CA, USA) was used in accordance with the manufacturer’s instructions. The absorbance was measured using a spectrophotometer at a wavelength of 450 nm. The BrdU proliferation assays were repeated at least three times, and each sample was measured in at least triplicate.

### Immunofluorescence

Cells were cultured on poly-D-lysine-coated 0.17 mm coverslips (Gerhard Mentzel GmbH, Braunschweig, Germany). These cells were colonized with lactobacilli for 24 hours, carefully washed, and then fixed for 10 minutes in 3.7% paraformaldehyde (PFA). Cells were permeabilized for 5 minutes in 20 mM Tris-HCl at pH 7.4, 50 mM NaCl, 3 mM MgCl_2_, 0.5% Triton X-100, and 300 mM sucrose and blocked with 1% skim milk powder (SMP) for 1 hour at room temperature. Rabbit anti-p21 (H-164, Santa Cruz Biotechnology Inc., Santa Cruz, CA, USA, 1∶500 dilution) and goat anti- rabbit IgG-Alexa 488 nm (Invitrogen, 1∶500 dilution) antibodies were used to detect p21.

### Flow Cytometry

Lactobacilli were added to non-confluent ME-180 cells. Unbound bacteria were removed after 2 hours of incubation, and the cells were incubated for an additional 22 hours. The cells were washed in PBS and detached by the addition of 0.2% trypsin-EDTA (Invitrogen, Carlsbad, CA, USA). The cells were collected by centrifugation at 778×*g* for 10 min at 4°C. The supernatant was decanted, and the cells were fixed in ice-cold 70% ethanol at 4°C. For the analysis, the cells were washed in PBS, treated with RNase (40 µg/ml, Sigma-Aldrich, St. Louis, MO, USA) and stained with propidium iodide (40 µg/ml, BD Biosciences) for 30 min at 37°C. The total DNA profile was generated by flow cytometry (FACSCalibur), and the data were analyzed using the Cell Quest Pro software package (BD Biosciences, Franklin Lakes, NJ, USA). Flow cytometry experiments were performed four times in at least duplicate, and for each sample, 10,000 events were recorded.

### Quantitative Real-time PCR Analysis

Lactobacilli were added to non-confluent ME-180 cells. Unbound bacteria were removed after 2 hours of incubation, and the cells were incubated for an additional 22 hours. The cells were washed in PBS, and RNA was isolated with an RNeasy Mini Kit in accordance with the manufacturer’s recommendations (Qiagen, Hilden, Germany). The total RNA (up to 1 µg) from three independent experiments was reverse transcribed using the Transcriptor First Strand cDNA Synthesis Kit (Roche, Basel, Switzerland); oligo-dT primers were employed for this process in accordance with the manufacturer’s recommendations. PCR amplification was performed using a LightCycler 480 (Roche, Basel, Switzerland) and a LightCycler 480 SYBR Green I Master kit (Roche, Basel, Switzerland) with 0.1 µM of each gene-specific primer and 1 µl of cDNA. The primers that were used were as follows: the CDKN1A forward primer, 5′-TGA GCT GCG CCA GCT GAG GT-3′; the CDKN1A reverse primer, 5′-TGC CGC ATG GGT TCT GAC GG-3′; the TUBA1A forward primer, 5′-ACA TCG ACC GCC TAA GAG TCG C-3′; and the TUBA1A reverse primer, 5′-TGC ACT CAC GCA TGG TTG CTG-3′. The following PCR program was employed: an initial denaturation at 95°C for 300 s, then 40 cycles of amplification involving denaturation at 95°C for 20 s, annealing at 60°C for 15 s and extension at 72°C for 15 s. The transition rate was 2.2–4.4°C/s. A melting curve analysis was conducted in three segments: 95°C for 5 s, 70°C for 60 s and a subsequent increase to 95°C (at a transition rate of 0.19°C/s). The transition rate for the first and second segment of this melting curve analysis was 2.2–4.4°C/s. The relative expression levels of the mRNAs of the target genes were calculated, normalized, and compared between control cells and infected cells, using alpha-tubulin (*TUBA1A*) and glyceraldehyde-3-phosphate dehydrogenase (*GAPDH*) as standards. Materials and methods for BAX analysis and MTT analysis are described in the supporting information section.

### Statistical Analyses

One-way ANOVA was used to evaluate whether the means that were obtained for treated and untreated cells differed in both the FACS assays and the BrdU assays. The statistical significance was determined with post hoc Dunnett’s multiple comparison tests (p<0.05). The GraphPad Prism 5.0 software package was used for statistical analysis. A paired 2-tailed Student’s *t*-test was used to evaluate the statistical significance of the qPCR data and the time-lapse data. All of the experiments were performed at least in triplicate, and the error bars that are provided in the figures represent the standard deviations.

## Results

### Lactobacilli Decelerate the Host Cell Cycle

**Figure 1:**
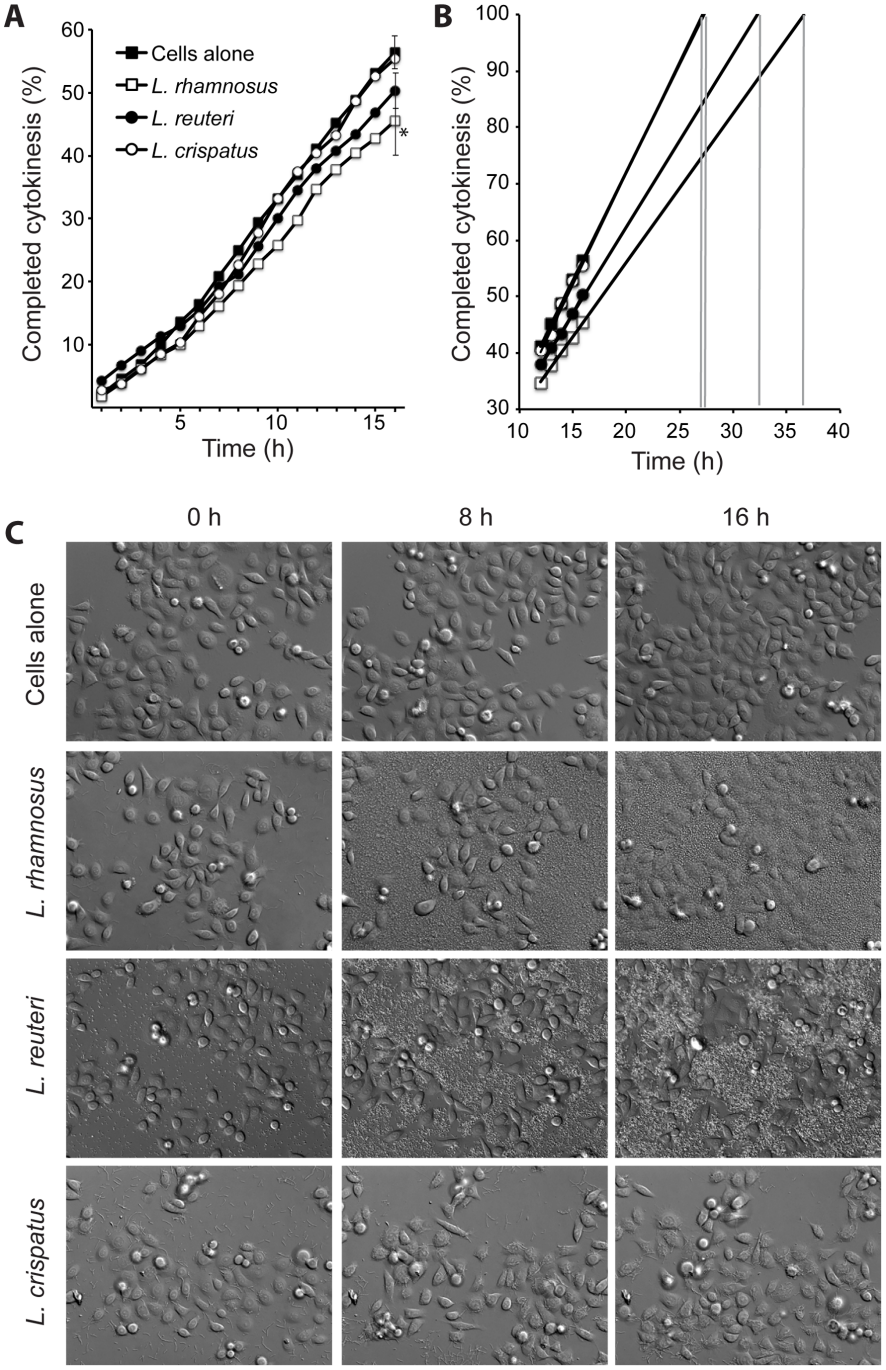
Host cell cycle proliferation was decelerated by the colonization of *L.* rhamnosus** or *L. reuteri* but not *L. crispatus*. Lactobacilli were allowed to colonize a non-confluent layer of ME-180 cells at 37°C in a 5% CO_2_ environment for 16 hours. DIC images were captured at multiple positions every 10 minutes throughout the live-cell microscopy. The percentage of cells that visually completed cytokinesis over time is displayed for both ME-180 cells that have been cultured alone, and ME-180 cells that have been colonized with one of the three *Lactobacillus* strains (A). The graph illustrates the hypothetical progression toward the time of a completed cell cycle for both ME-180 cells alone and ME-180 cells that have been colonized with each of the three *Lactobacillus* strains (B). Representative DIC images of both ME-180 cells alone and ME-180 cells that have been colonized with each of the three *Lactobacillus* strains, captured at the start (0 hours), midpoint (8 hours), and termination (16 hours) of the experimental procedures (C). Standard deviations for the 16-hour time-point experiments are shown (*indicates that *p*<0.05).

**Figure 2:**
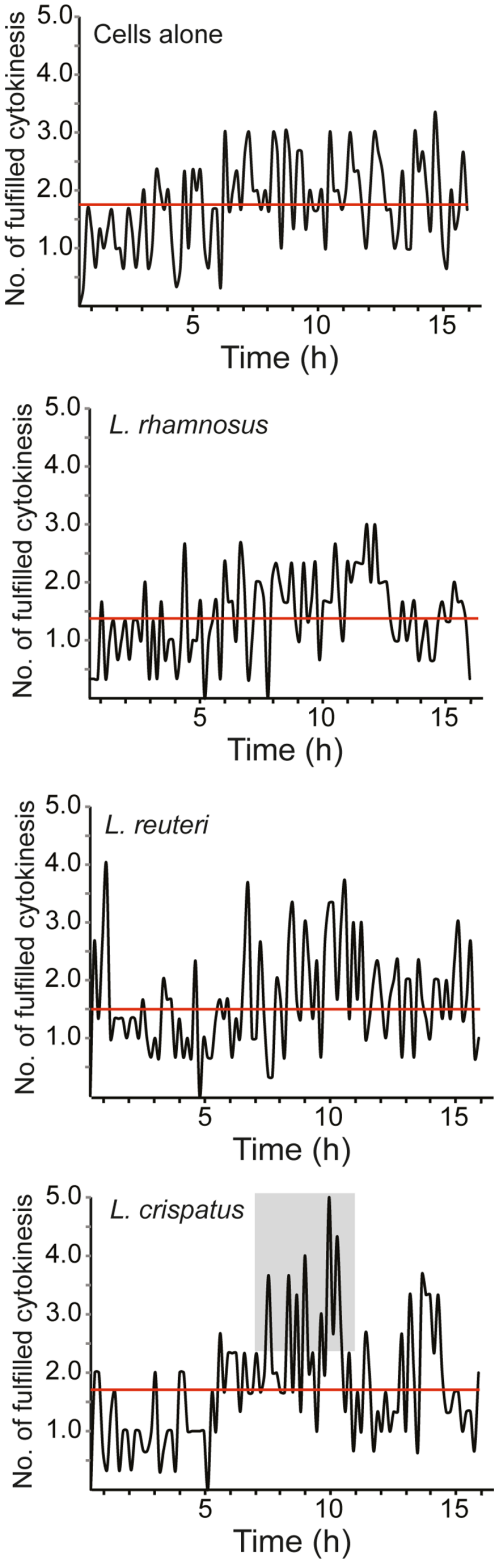
***L.*** *crispatus* transiently increases cell division frequency. Lactobacilli were allowed to colonize a non-confluent layer of ME-180 cells at 37°C in a 5% CO_2_ environment for 16 hours. DIC images were captured at multiple positions every 10 minutes. The number of cell division events that occurred every 10 minutes during the 16 hours of the assay is shown for both ME-180 cells alone, and ME-180 cells that have been colonized with each of the three *Lactobacillus* strains. The gray box highlights the increased cell division frequency that was observed. The red line indicates the average cell division frequency.

The three different *Lactobacillus* strains, *L. rhamnosus, L. reuteri* and *L. crispatus*, were allowed to adhere to ME-180 cells separately. The cell cycle progression was monitored by live cell microscopy; in particular, images were acquired every 10 minutes for a total of 16 hours. A total of 900 individual cells per assay were followed, and the time point when each cell had completed cytokinesis was recorded. After 16 hours, 56% of the uninfected ME-180 cells had completed cytokinesis. *L. rhamnosus* and *L. reuteri* affected the ME-180 cells by decreasing the cell cycle rate, producing the result that only 45% and 50%, respectively, of the ME-180 cells completed cytokinesis during the 16 hours of microscopy (**Fig. 1A**). By contrast, 55% of the ME-180 cells that were colonized by *L. crispatus* had completed cytokinesis after 16 hours; thus, these cells did not differ from untreated ME-180 cells with respect to cell cycle progression. Time points of *Lactobacillus* colonization that were longer than 16 hours were not feasible, because the dense layer of bacteria that covered the epithelial cells at colonization durations that were longer than 16 hours produced an impaired ability to visually monitor all of the cytokinesis events (data not shown). The standard deviations between control cells and cells colonized with either *L. rhamnosus* or *L. reuteri* do not overlap, but the values at 16 hours time-point have high standard deviations, due to the ability of the bacterial colonization to influence cell division frequencies of the host target cells. Standard deviations were intentionally shown only in the 16 hours time-point in figure 1A since the true variance of fulfilled cytokinesis over the complete time period is presented in figure 2.

No increased apoptosis rates were visually observed. This was confirmed by a MTT cell viability assay and a qPCR assay on the pro-apoptotic gene BAX (**Fig. S1**). Then, we extrapolated from the 11–16 hour time points; based on a linear progression, the time that would be required for 100% of the cells to complete cytokinesis. Using this assumption, 100% of ME-180 cells alone would have completed cytokinesis after 27 hours (**Fig. 1B**). This correlated well with 72-hours time-lapse data on ME-180 cells, where individual cells were followed for timing the cell cycle (data not shown). Cells that were colonized with *L. crispatus* would exhibit an average completion time of 27.5 hours, a difference of only 30 minutes from the control ME-180 cells. *L. rhamnosus* and *L. reuteri* colonization would delay the average completion time to 37 hours and 32.5 hours, respectively.

The time-lapse experiments began at a low cellular confluence. Figure 1C presents representative images from the start (0 h), midpoint (8 h), and end (16 h) of the time-lapse experiments, illustrating how the different lactobacilli adhere to and colonize ME-180 cells and how the cells progress through the cell cycle during the course of the assay. Despite the unique morphological differences in the lactobacillus strains and the ability of the bacteria to bind to both the ME-180 cells and the cell culture dish, space remained available in the cell culture for the cells to divide (**Fig. 1C** **, Movie S1, S2, S3, S4**).

In combination, these data indicate that *L. rhamnosus* and *L. reuteri* colonization caused a cell cycle deceleration in ME-180 cells, theoretically prolonging the cell cycle by 5–10 hours.

### The Frequency of Cell Division Differs among the Lactobacillus Strains

During the observations of 900 cells by live-cell microscopy, it came to our attention that for certain strains, the number of cells in late mitosis was not equally distributed over the 16 hours of the assay. Subsequently, the number of completed cytokinesis events that was observed in every timeframe of 10 minutes for the duration of 16 hours was analyzed. For an average of 900 cells that were followed during the 16-hour assay, the ME-180 cells alone performed 1.7 cell divisions every 10 minutes (**Fig. 2**). Colonization with *L. rhamnosus*, and *L. reuteri* produced lowered cell division frequencies of 1.4 and 1.5 cell divisions every 10 minutes, respectively, over the same time period (**Fig. 2**). By contrast, cells that were colonized with *L. crispatus* shifted their modes of cell division frequency between 7 and 11 hours; at those times, these cells exhibited an average of 2.3 cell divisions every 10 minutes with peaks of up to 5 cell divisions every 10 minutes (**Fig. 2**). However, the increased number of cell divisions that was induced by *L. crispatus* did not alter the overall average proliferation rate. These data demonstrate the ability of *L. crispatus* to transiently enhance the frequency of cell division.

### S phase Progression is Reduced by *L. rhamnosus* and *L. reuteri* but not *L. crispatus*

**Figure 3:**
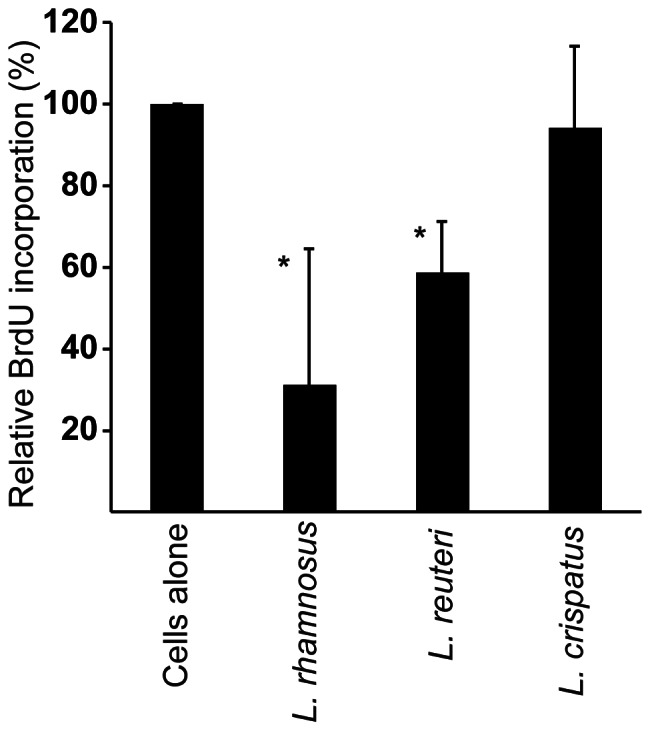
*L.* *rhamnosus* and *L. reuteri* but not *L. crispatus* reduced S-phase progression in ME-180 cells. The relative BrdU incorporation of ME-180 cells that were colonized for 24 hours with each of the three *Lactobacillus* strains was measured. The BrdU incorporation for the ME-180 cells alone was set to 100%. The means and standard deviations from 6 experiments are shown (*indicates that *p*<0.05).

To further verify the finding that *Lactobacillus* colonization reduces cell cycle progression, the number of cells in S phase at the end of 24 hours of incubation was measured. Lactobacilli were allowed to adhere to ME-180 cells for 24 hours. Bromodeoxyuridine (BrdU) was added to cells two hours prior to the termination of this assay, allowing the BrdU to be incorporated into the replicating DNA of cells in S phase. The adherence by all of the lactobacillus strains that were tested except for *L. crispatus* resulted in a reduced number of ME-180 cells in S phase. *L. rhamnosus* and *L. reuteri* reduced the level of BrdU incorporation to an average of 31% and 59%, respectively, relative to the BrdU incorporation level of control ME-180 cells (**Fig. 3**). Thus, this finding once again demonstrates that colonization with *L. rhamnosus* and *L. reuteri* but not *L. crispatus* inhibits cell cycle progression in ME-180 cells.

### Lactobacilli Delay ME-180 Cells in G1 Phase

**Figure 4:**
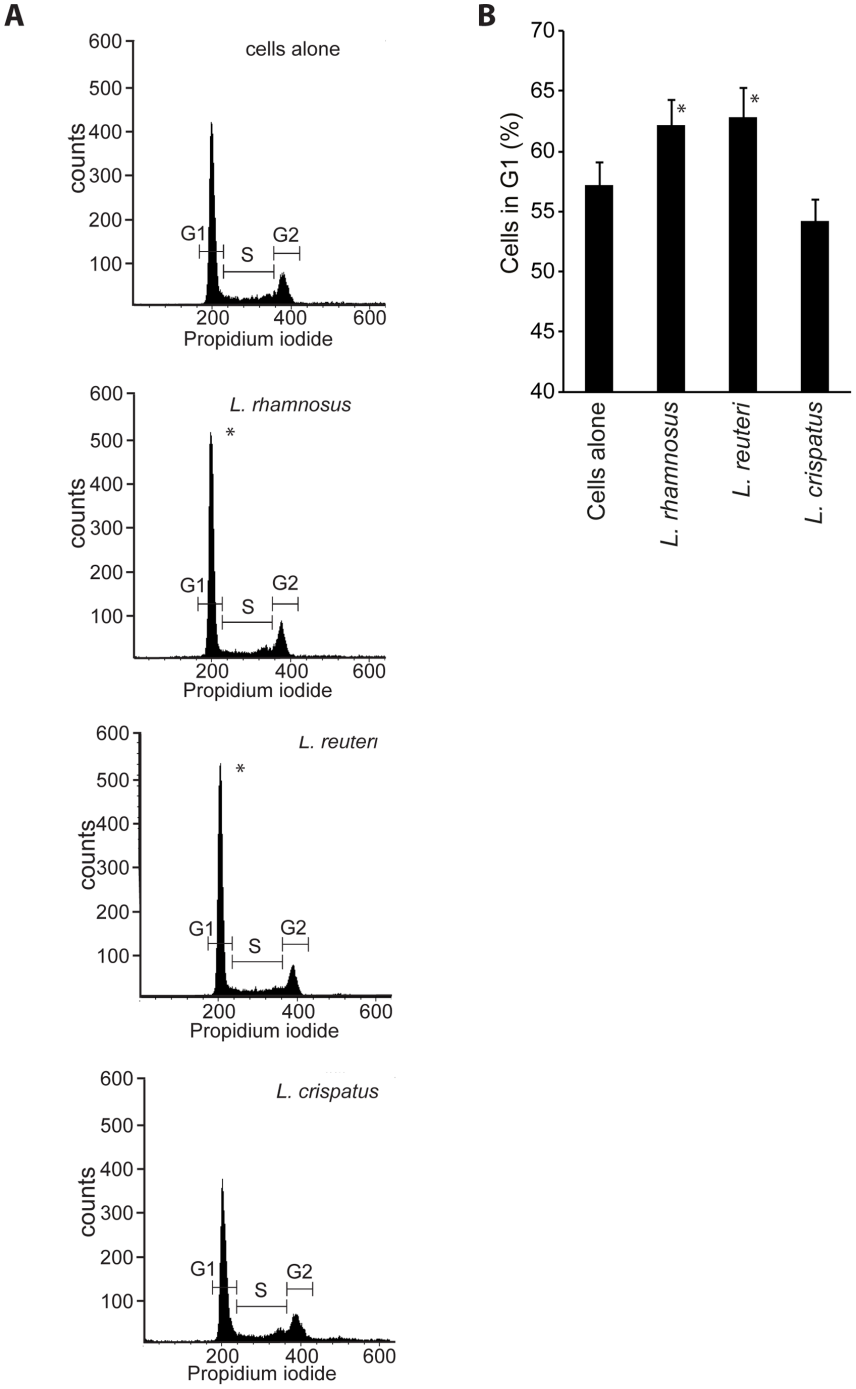
*L.* *rhamnosus* and *L. reuteri* cause ME-180 cells to accumulate in the G1 phase of the cell cycle. Lactobacilli were added to ME-180 cells and incubated for 24 hours. The cells were fixed in 70% ethanol, treated with RNase and stained with propidium iodide. The total DNA profile of 10 000 cells was analyzed by flow cytometry. Representative histograms of both ME-180 cells alone and ME-180 cells that have been colonized with each of the three *Lactobacillus* strains are shown (A). The number of cells situated in G1 phase was significantly higher in both *L. rhamnosus* and *L. reuteri* colonized cells (B). Four independent experiments were analyzed (*indicates that *p*<0.05).

To determine whether the bacterial-induced cell cycle delay occurred during a specific cell cycle phase, we analyzed cell phase profiles by flow cytometry. Bacteria were added to ME-180 cells for 24 hours, and cellular DNA was stained with propidium iodide. Four independent experiments were performed, and the percentages of cells in G1, S, and G2 were quantified. Representative flow cytometry histograms of the control ME-180 cells and the ME-180 cells that were colonized with the three *Lactobacillus* strains are presented in figure 4A. For the control ME-180 cells, an average of 57% of the examined cells were in G1 phase. Adhesion by *L. rhamnosus* and *L. reuteri* significantly increased the number of cells in G1 phase to an average of 62% and 63%, respectively (Fig. 4B). *L. crispatus* colonization did not significantly alter the number of cells in G1 phase (54%).

### *L. rhamnosus* Colonization Up-regulates p21

**Figure 5:**
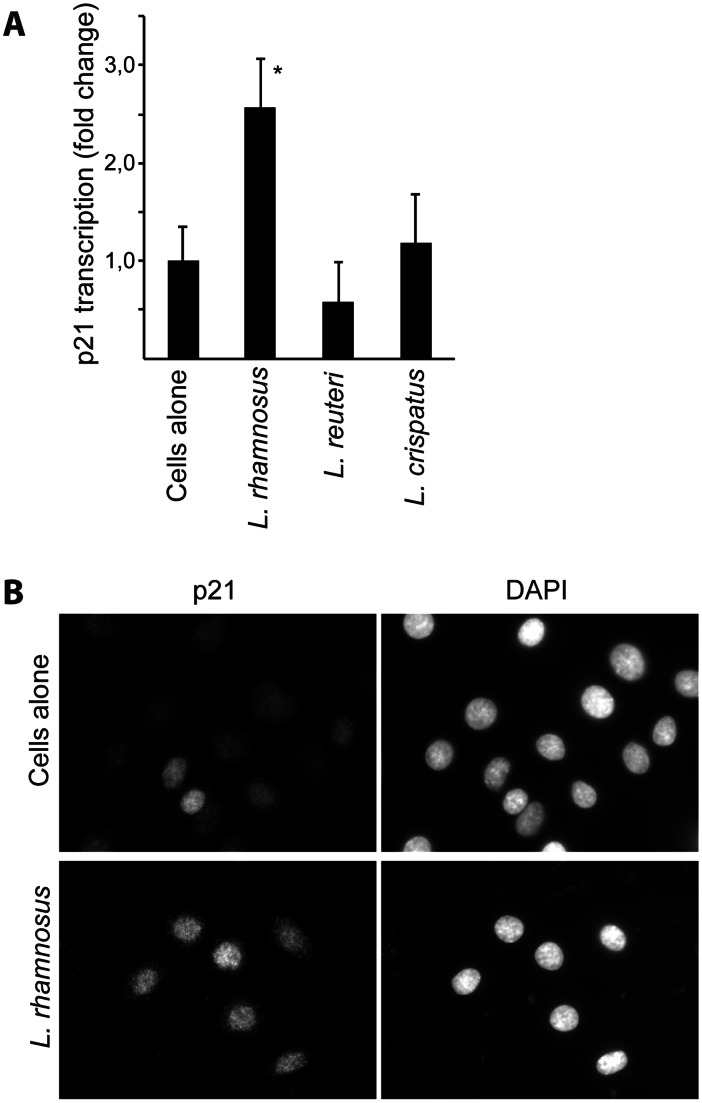
*L.* *rhamnosus* up-regulates and redistributes p21 in ME-180 cells. Lactobacilli were allowed to colonize a non-confluent layer of ME-180 cells for 24 hours. The cells were prepared for qPCR analysis or immunofluorescence. The graph shows the normalized relative p21 mRNA levels in ME-180 cells upon colonization by the three different lactobacillus strains. Three independent experiments were analyzed (*indicates that *p*<0.05) (A). Immunofluorescence images displaying the p21 protein localization and expression for both ME-180 cells alone and ME-180 cells that have been colonized with *L. rhamnosus* for 24 hours. The cellular DNA was also stained with DAPI. The scale bar represents 10 µm (B).

The G1 accumulation of cells is often mediated by increases in the activity of cyclin-dependent kinase inhibitors, such as p21. To investigate the role of p21 in lactobacillus-mediated cell cycle deceleration, lactobacilli were allowed to adhere to ME-180 cells for 24 hours. Bacteria-colonized cells were washed and prepared for either qPCR assay or immunofluorescence staining. Through the use of p21-specific primers for qPCR and the normalization of the results against the mRNA levels of α-tubulin (**Fig. 5A**) or GAPDH (data not shown), we demonstrate that *L. rhamnosus* up-regulated p21 mRNA 2.5-fold in comparison to control cells. No significant change in p21 mRNA levels was observed in either *L. reuteri* or *L. crispatus*. The p21 protein is a cell cycle inhibitor, and its nuclear localization is associated with a slowing of the cell cycle [12], [13]. Indeed, compared with the control ME-180 cells, *L. rhamnosus*-colonized cells displayed a greater extent of p21 expression in their cellular nuclei (**Fig. 5B**).

To summarize, *L. rhamnosus* colonization up-regulates p21 in ME-180 cells; this up-regulated p21 is localized in the cellular nucleus.

### The Secretion of Lactic Acid Reduces Cell Cycle Progression

**Figure 6:**
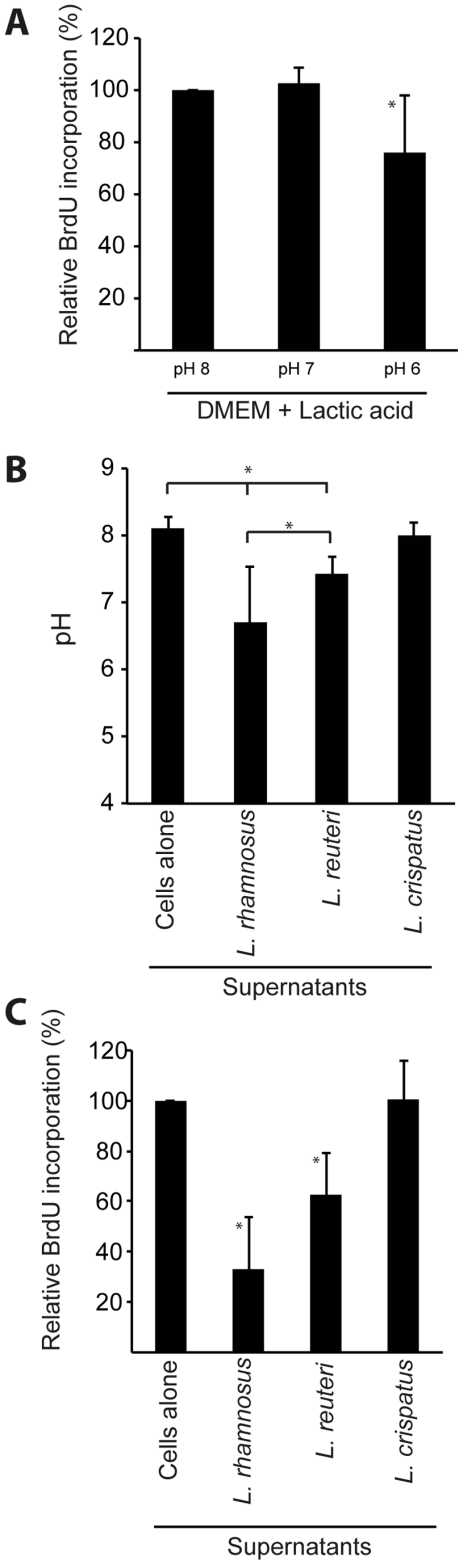
The secretion of lactic acid reduces cell cycle progression. The relative BrdU incorporation was measured in ME-180 cells in DMEM that has been adjusted with lactic acid to pH 7 or pH 6. The BrdU incorporation in DMEM (pH 7.4) was set to 100%. The means and standard deviations from five experiments are shown. (*indicates that *p*<0.05) (A). The graph indicates the pH levels that were measured in cell culture supernatants after ME-180 cells underwent 24 hours of incubation either alone or colonized with lactobacilli. The average pH values and standard deviations from 6 independent experiments are shown (and *indicates that p<0.05) (B). The relative BrdU incorporation of ME-180 cells that have been exposed for 24 hours to sterile-filtered cell culture supernatants originating from 24 hours of incubation of ME-180 cells either alone or colonized with lactobacilli. The BrdU incorporation level of the cells incubated with the supernatant from ME-180 cells alone was set to 100%. The averages and standard deviations from 3 independent experiments are shown (*indicates that p<0.05) (C).

When ME-180 cells were colonized with the different *Lactobacillus* strains, the cell media underwent a reduction in pH due to the lactic acid production by the bacteria. To investigate whether the pH change alone could affect the cell cycle, we adjusted the DMEM (normally pH 7.4) with lactic acid to pH 7 and pH 6 and added this medium to ME-180 cells for 24 hours. Although the same amounts of lactic acid were added in three different experiments (in triplicate), the pH varied during the BrdU incorporation due to the buffering capacity of the cell culture medium. The pH 6 treatments significantly reduced cell proliferation, producing a relative BrdU incorporation of 76% relative to control cells; this pH is achieved through the addition of 2.7 mg/ml of lactic acid (**Fig. 6A**). No reduction in cell proliferation was observed with cell culture medium that was adjusted to pH 7.

Next, we measured the pH of the cell supernatants after 24 hours of colonization by lactobacilli. The ME-180 cells alone displayed an average of pH of 8.1, and colonization by *L. rhamnosus* caused a pH reduction to 6.7 in the cell supernatant, whereas *L. reuteri* (pH 7.4) and *L. crispatus* (pH 8.0) caused only moderate reductions in the cell culture pH (**Fig. 6B**). Thus, only *L. rhamnosus* was able to reduce the supernatant pH to levels approaching pH values known to decrease cell proliferation rates. Subsequently, the 24-hour supernatants from the colonized cells were sterile-filtered and transferred to a new set of cell culture dishes of ME-180 cells, and the S phase progression of these cells was investigated for 24 hours. Supernatants were diluted 1∶2 in fresh medium to avoid possible nutrient reduction. Supernatants from cells colonized with *L. rhamnosus* reduced the new cells to a BrdU incorporation of 28% relative to control cells (**Fig. 6C**). In addition, the *L. reuteri* supernatant reduced the number of cells progressing through S phase. As expected, compared with control cells, the supernatant from *L. crispatus* did not alter S phase progression. This observation indicates that pH levels that are lowered by lactic acid production, as occurred in the case of *L. rhamnosus* colonization, can inhibit cell cycle progression.

## Discussion

Epithelia are physical barriers and compose the interface between distinct body compartments and the outer world. The maintenance of an active cell cycle by epithelial cells enables these barriers to remain intact. The normal flora of an individual, and particularly the lactobacilli in the female genital tract, contribute to the mucosal environment by efficiently adhering to the epithelial cells and the mucus layer. In this study, we demonstrate that certain lactobacilli can also influence the cell cycle speed of the epithelial cells. ME-180 cervical cell cycle progression during colonization by three different *Lactobacillus* strains was studied. A cell cycle deceleration was observed in the BrdU assay from the exposure of cells to the gastric strain *L. rhamnosus* or the saliva strain *L. reuteri*, but not the vaginal strain *L. crispatus*. Based on a linear progression, we estimated that the bacterial colonization caused a 5-to-10-hour delay in the cell cycle; the actual delay may be more pronounced than this estimate, given that the deceleration was likely to continue for the time period between 16–27 hours of the total cell cycle. The same two *Lactobacillus* strains also reduced the number of cells going through S phase due to an accumulation of cells in G1 phase. Thus, lactobacilli can slow the cell cycle, primarily by halting the cells in G1 phase for a longer time. This finding is further confirmed by the fact that *L. rhamnosus* causes the up-regulation of p21, a potent CDK inhibitor that is known to be up-regulated upon cell cycle arrest, particularly during cell cycle arrest in G1 phase. The fact that only *L. rhamnosus* but not *L. reuteri* produced the up-regulation and subcellular redistribution of p21 may be due to different reasons. The higher production of lactic acid leading to lower pH levels during *L. rhamnosus* colonization or different production of bacteriocins may be the cause. Also, *L. rhamnosus* bind to a higher extent to the cells and may contribute to the contact inhibition signaling [11]. This interesting difference will be of value future research.

Lactobacilli in the vaginal tract help maintain a low pH through the production and secretion of lactic acid. We demonstrate that the addition of sufficient lactic acid to change the cell medium from pH 8 to pH 6 can reduce cell proliferation in cervical cells. However, only the gastric-isolated *L. rhamnosus* was able to produce an effect on cell proliferation through the sufficient reduction of the pH of the cell medium. By contrast, despite the fact that *L. acidophilus* reduces the cell medium to a pH of 4.5, this bacterial species has been reported to increase the proliferation of human vaginal epithelial cells [14]. Lactic has been shown to cause cell cycle arrest and apoptosis in human keratinocyte cells [15]. Future work must establish whether this effect, in which vaginal cells would be less affected by a lowered pH but cervical cells should suffer from reduced proliferation under acidic conditions, reflects a process that actually occurs in vivo; it is possible that the observed cell cycle delay is simply a cell line-dependent behavior.

Intriguingly, the supernatant from *L. reuteri*, although it caused only a moderate pH reduction, was still able to reduce host cell proliferation. This observation indicates that other factors are involved in the induction of the reduced proliferation. It is likely that the heavy colonization of cells by *L. reuteri*, which contrasts with the moderate colonization of cells by *L. crispatus,* mimics the cellular confluence signals on the epithelial cell surface, resulting in a slowdown of the proliferation speed. Due to their differences in lactobacillus morphology and aggregation, the coat over and/or around epithelial cells that different lactobacilli can form varies greatly. However, the epithelial monolayer beneath the carpet of lactobacilli appears healthy, and no increased apoptosis of the epithelial cells is observed. During experimental assays, great care was taken to perform all assays at low confluence, ensuring that cell-to-cell contact inhibition was never the cause for cell cycle arrest.

In combination, the results of this study demonstrate that the properties of each *Lactobacillus* strain are unique. Certain *Lactobacillus* strains have been found to possess anti-proliferative effects on human gastric and colon cancer cells [6], [7], [16]. In this study, we reveal the differences between *L. rhamnosus,* which possesses anti-proliferative qualities for cervical cells, and *L. crispatus*, which lacks this quality but has the ability to transiently increase the mitotic frequencies of the host cells. However, it appears reasonable that the delicate composition of the multiple *Lactobacillus* species colonizing the female genital tract would be able to regulate epithelial renewal, given that lactobacilli, as reported both in this study and in previous studies, can exert both anti-proliferative and proliferative effects on host cells.

## Supporting Information

Figure S1 **Lactobacilli were allowed to colonize a non-confluent layer of ME-180 cells for 24 hours.** The cells were prepared for MTT assay or qPCR. The graph shows the normalized MTT values in ME-180 cells upon colonization by the three different lactobacillus strains. The means and standard deviations from 4 experiments are shown (*indicates that *p*<0.05) (A). The lower graph shows the normalized relative BAX mRNA levels from two experiments (in duplicates) in ME-180 cells upon colonization by the three different lactobacillus strains (B).
(TIF)

Materials and Methods S1 (DOCX)

Movie S1 **Cell growth and proliferation of ME-180 cells.** ME-180 cells were grown and maintained in 35 mm poly-D-lysine-coated glass-bottom dishes for 16 hours in a live-cell incubator that was connected to an inverted microscope (Cell Observer, Carl Zeiss). The cells were maintained at 37°C in 5% CO_2_ throughout the microscopy process. DIC images were captured every 10 minutes. The image sequences were further processed into a movie by ImageJ software.
(AVI)

Movie S2 **Cell growth and proliferation of ME-180 cells upon colonization with** ***L. rhamnosus*** **.** ME-180 cells, grown and maintained in 35 mm poly-D-lysine-coated glass-bottom dishes were challenged with lactobacilli for 16 hours of incubation. At the start of the assay, cells were washed once and then transferred to a live-cell incubator that was connected to an inverted microscope (Cell Observer, Carl Zeiss). The cells were maintained at 37°C in 5% CO_2_ throughout the microscopy process. DIC images were captured every 10 minutes. The image sequences were further processed into a movie by ImageJ software.
(AVI)

Movie S3 **Cell growth and proliferation of ME-180 cells upon colonization with** ***L. reuteri*** **.** ME-180 cells, grown and maintained in 35 mm poly-D-lysine-coated glass-bottom dishes were challenged with lactobacilli for 16 hours of incubation. At the start of the assay, cells were washed once and then transferred to a live-cell incubator that was connected to an inverted microscope (Cell Observer, Carl Zeiss). The cells were maintained at 37°C in 5% CO_2_ throughout the microscopy process. DIC images were captured every 10 minutes. The image sequences were further processed into a movie by ImageJ software.
(AVI)

Movie S4 **Cell growth and proliferation of ME-180 cells upon colonization with** ***L. crispatus*** **.** ME-180 cells, grown and maintained in 35 mm poly-D-lysine-coated glass-bottom dishes were challenged with lactobacilli for 16 hours of incubation. At the start of the assay, cells were washed once and then transferred to a live-cell incubator that was connected to an inverted microscope (Cell Observer, Carl Zeiss). The cells were maintained at 37°C in 5% CO_2_ throughout the microscopy process. DIC images were captured every 10 minutes. The image sequences were further processed into a movie by ImageJ software.
(AVI)
