# Supplementary figures and images for: Lactobacillus Decelerates Cervical Epithelial Cell Cycle Progression
Source: PLoS One. 2013 May 10;8(5):e63592. doi: 10.1371/journal.pone.0063592 (PMC3651132; doi:10.1371/journal.pone.0063592)

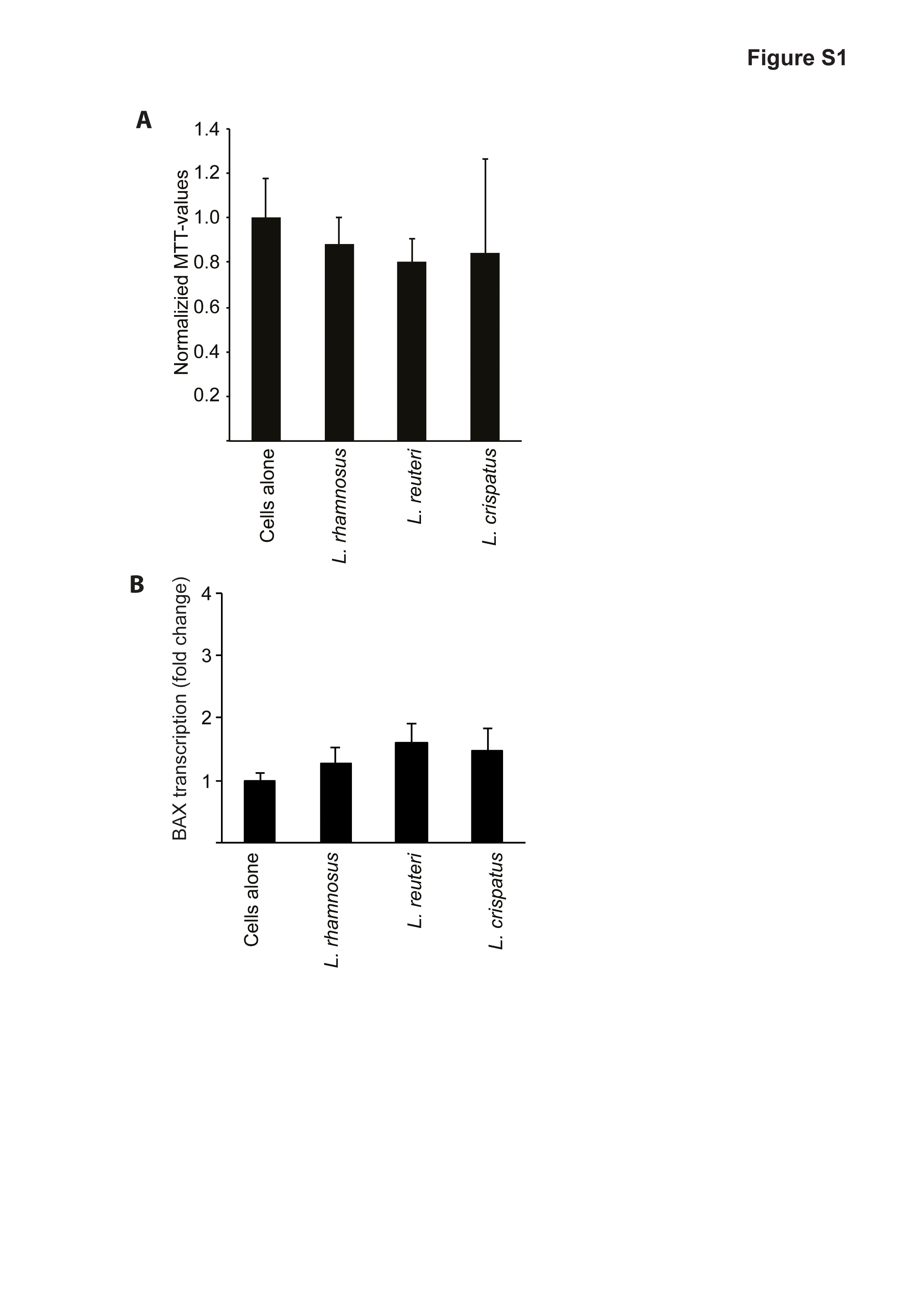

Supplement: Figure S1 — Lactobacilli were allowed to colonize a non-confluent layer of ME-180 cells for 24 hours. The cells were prepared for MTT assay or qPCR. The graph shows the normalized MTT values in ME-180 cells upon colonization by the three different lactobacillus strains. The means and standard deviations from 4 experiments are shown (*indicates that p<0.05) (A). The lower graph shows the normalized relative BAX mRNA levels from two experiments (in duplicates) in ME-180 cells upon colonization by the three different lactobacillus strains (B). (TIF) [file pone.0063592.s001.tif]
