# Supplementary material for: Lactobacillus Decelerates Cervical Epithelial Cell Cycle Progression
Source: PLoS One. 2013 May 10;8(5):e63592. doi: 10.1371/journal.pone.0063592 (PMC3651132; doi:10.1371/journal.pone.0063592)
Supplement: Materials and Methods S1 — (DOCX) [file pone.0063592.s002.docx]

**Supplementary materials and methods**

*MTT assay*

Bacteria were added to the low-confluent monolayer of ME-180 cells (m.o.i. of 50) and allowed to adhere for 2 hours, after which the unbound bacteria were removed and the cells incubated further. Cells were infected with lactobacilli for a total of 24 hours. MTT (3-(4,5-[Di](http://en.wikipedia.org/wiki/Di-)[methyl](http://en.wikipedia.org/wiki/Methyl)[thiazol](http://en.wikipedia.org/wiki/Thiazole)-2-yl)-2,5-di[phenyl](http://en.wikipedia.org/wiki/Phenyl)tetrazolium bromide was added to the cells at a final concentration of 5 mg/ml and incubated for 20 minutes at 37° C, 5 % CO_2_. The supernatant was removed completely and 0.2 ml dimethyl sulfoxide was added for 10 minutes in 37°C. The absorbance was measured using a spectrophotometer at a wavelength of 540 nm. The MTT assay were repeated at least three times, and each sample was measured in at least triplicate

*Quantitative real time PCR analysis*

Bacteria were added to the non-confluent monolayer of ME-180 cells (m.o.i. of 50) and allowed to adhere for 2 hours, after which the unbound bacteria were removed and the cells incubated further. Cells were infected with lactobacilli for a total of 24 hours. RNA from control cells and infected cells was isolated with the RNeasy Mini kit according to manufacturers recommendations (Qiagen, Hilden, Germany). Total RNA (up to 1 μg) was reversed transcribed using Transcriptor First Strand cDNA synthesis kit (Roche, Basel, Switzerland), using oligo-dT primers according to manufacturers recommendations. Primers used were BAX forward: 5’- TCG GGG ACG AAC TGG ACA GTA A -3’, BAX reversed: 5’- ATG TCA GCT GCC ACT CGG AAA A -3’, TUBA1A forward: 5’- ACA TCG ACC GCC TAA GAG TCG C -3’, TUBA1A reversed: 5’- TGC ACT CAC GCA TGG TTG CTG -3’ and GAPDH forward: 5’- TCG TCA TGG GTG TGA ACC ATG AGA -3’, GAPDH reversed: 5’- TGT GGT CAT GAG TCC TTC CAC GAT -3’. PCR amplification was performed using a Light Cycler 480 (Roche) and SYBR Green I Master kit (Roche) with 0.1µM of each gene specific primer and 5µl of cDNA. PCR program was as follows: initial denaturation for 300 s, amplification for 30 cycles with denaturation at 94°C for 30 s, annealing at 55°C for 15 s and extension at 71°C for 30 s. The transition rate was 2.2 - 4.4°C/s. Relative expression of mRNA of the target genes were calculated, normalized, and compared between control cells and infected cells, using alpha tubulin (*TUBA1A*) as an internal standard and Glyceraldehyde 3-phosphate dehydrogenase (*GAPDH*) as an additional standard.
